# Evaluating seven bioinformatics platforms for tertiary analysis of genomic data from whole exome sequencing in a pilot group of patients

**DOI:** 10.1515/almed-2025-0031

**Published:** 2025-03-10

**Authors:** Nerea Bastida-Lertxundi, Itxaso Martí-Carrera, Borja Laña-Ruíz, Otilia Martínez-Múgica Barbosa, Raquel Muguerza-Iraola, Raquel Sáez-Villaverde, Julien S. Crettaz

**Affiliations:** Biogipuzkoa Health Research Institute, Neurogenetics, Biology and RNA Therapies Research Group – NeuroRNA, San Sebastián, Spain; Osakidetza, Donostialdea Integrated Health Organization, Unit of Clinical Genetics, Donostia University Hospital, San Sebastián, Spain; Biogipuzkoa Health Research Institute, Research Group in Paediatrics, San Sebastián, Spain; Osakidetza, Donostialdea Integrated Health Organization, Unit of Paediatrics, Donostia University Hospital, San Sebastián, Spain; Department of Paediatrics, University of the Basque Country UPV/EHU, San Sebastián, Spain

**Keywords:** automated prioritization, automated classification, artificial intelligence, whole exome, tertiary analysis, bioinformatics tool

## Abstract

**Objectives:**

To evaluate seven bioinformatics platforms for automated AI-based genomic variant prioritization and classification.

**Methods:**

An evaluation was performed of 24 genetic variants that explained the phenotype of 20 patients. FASTQ files were simultaneously uploaded on the following bioinformatics platforms: Emedgene, eVai, Varsome Clinical, CentoCloud, QIAGEN Clinical Insight (QCI) Interpret, SeqOne and Franklin. Automated variant prioritization and classification was performed using patient phenotypes. Phenotypes were entered onto the different platforms using HPO terms. The classification of reference was established based on the criteria of the American College of Medical Genetics and Genomics (ACMG) and the Association of Molecular Pathology and ACMG/ClinGen guidelines.

**Results:**

SeqOne demonstrated the highest performance in variant prioritization and ranked 19 of 24 variants in the Top 1; four in the Top 5, and one in the Top 15, followed by CentoCloud and Franklin. QCI Interpret did not prioritize six variants and failed to detect one. Emedgene did not prioritize one and failed to detect one. Finally, Varsome Clinical did not prioritize four variants. Franklin classified correctly 75 % of variants, followed by Varsome Clinical (67 %) and QCI Interpret (63 %).

**Conclusions:**

SeqOne, CentoCloud, and Franklin had the highest performance in automated variant prioritization, as they prioritized all variants. In relation to automated classification, Franklin showed a higher concordance with the reference and a lower number of discordances with clinical implications. In conclusion, Franklin emerges as the platform with the best overall performance. Anyway, further studies are needed to confirm these results.

## Introduction

Estimations indicate that 5.9 % of the population is affected by a rare disease. Over 50 % of these patients will never have a final diagnosis [1], 2]. Patients with an uncertain diagnosis usually go through the so-called ‘diagnostic Odyssey’, a process that involves consultations with multiple specialists, numerous medical imaging studies and a myriad of clinical laboratory tests [3]. Molecular diagnosis contributes to improving disease management and optimizing treatments and follow-up. This approach opens the way to the delivery of genetic counselling to patients and their family regarding potential complications, risk of relapse and reproductive options [4].

Whole exome sequencing (WES) has been a breakthrough in genetic diagnostics. This technique allows for the parallel sequencing of 22,000 coding genes accounting for 2 % of the entire genome and containing 85 % of disease-causing variants [5], 6]. High-throughput platforms enable the sequencing of several exomes in a few hours at a reasonable cost and with a significant accuracy [7]. The genetics industry is currently facing the era of genomic data management. Massive data storage may be challenging firstly due to security, privacy and traceability issues. Secondly, finding a genetic cause may be like seeking a needle in a haystack [8], 9]. The WES of a subject comprises an average of 100,000 variants. In the clinical setting, screening for, prioritizing, interpreting and classifying clinically relevant variants is the most challenging stage of genetic diagnostics and is the bottleneck of genetic testing. Prior to the analysis of candidate variants, the datasets generated by sequencing platforms undergo several bioinformatic processing steps. At this stage, clinicians rely on the use of commercially available platforms. These platforms perform a secondary analysis of genomic data to identify variants not aligned with the reference genome [10], 11]. Next, these platforms perform a tertiary analysis that involves annotating each variant and collecting relevant information that is either intrinsic to the variant or available in research databases and in the scientific literature [12], [13], [14], [15], [16], [17]. Finally, variants are filtered and prioritized.

Until recently, the last step required a visual inspection of a list of variants displayed in tables using basic filters. In addition, variants used to be classified manually following the criteria provided in clinical guidelines [18], 19]. As a result, identifying the causative variant was challenging and time-consuming.

For an optimal tertiary analysis, patient details such as age, sex, phenotype and date of onset of symptoms, among other data, can be entered into these platforms. HPO terms (after ‘*human phenotype ontology’*) have become essential to this process, as they provide standardized terms for describing patient phenotypes. The use of HPO enables platforms to link patient HPO terms with their associated genes and related diseases, along with other parameters during the annotation process [20].

The great novelty of these bioinformatics BI platforms is that they are based on machine learning algorithms that generate artificial neural networks (ANNs). These networks correlate genotypes, genes, diseases, population databases, variant-phenotype databases, patient predictors and symptoms, among other parameters, and consider the scientific literature available. Moreover, ANNs perform automated variant prioritization in a fast and efficient way. These platforms integrate the criteria of the American College of Medical Genetics and Genomics (ACMG)/Association of Molecular Pathology (AMP) and ACMG/ClinGen guidelines [18], 19]. This capability makes it possible to automatically prioritize and classify variants.

The application of AI to clinical genomics will boost the implementation of WES in clinical practice and improve patient access to this technology. This step forward will certainly revolutionize healthcare [21].

To date, the literature available comparing variant prioritization tools is limited. Most of the papers published only evaluate open-access tools and use a phenotype-based filtering and prioritization approach. In addition, only one of them was based on IA-based algorithms [22], [23], [24], [25], [26], [27], [28], [29].

The objective of this study was to perform an objective, independent evaluation of different commercially-available BI platforms for the interpretation of genomic data.

The ultimate purpose of this project is to implement WES in the clinic. For such purpose, it is necessary that objective data is available for selecting the most effective tool. Access to a high-performing prioritization and classification tool will help clinical laboratory professionals reduce the time required for analysis and the associated costs, improve turnaround times, increase diagnostic yield and meet the growing demand for this type of genomic studies [30], 31].

## Materials and methods

A retrospective WES study was carried out to randomly select 20 patients with one or several variants related to their phenotype. These patients had visited different Units of Donostia University Hospital.

The diversity of patients enabled us to include a broad variety of deleterious variants and inheritance patterns. A total of 24 genetic variants were included and classified by the external laboratory. Later, these variants were inspected by professionals from the Unit of Clinical Genetics of Donostia University Hospital (UCG-DUH), who assigned the classification that would be used as reference (see Supplementary Material, Table 1 and Table 2). The classification of reference was established based on the criteria and recommendations of ACMG/AMP and ACMG/ClinGen guidelines [18], 19].

Six copy number variations (CNVs) were included. The length of deletions and duplications ranged from 1.9 kb to 9.4 Mb. All followed an autosomal dominant inheritance pattern, except for an X-linked deletion (see Supplementary Material, Table 3). 12 single nucleotide variants (SNV) were included, of which five were missense, four were nonsense, two were canonical splice site mutations and one was a silent mutation. Five small frameshift deletions and a small frameshift duplication were also included. These 18 variants had heterogeneous inheritance patterns (see Supplementary Material, Table 4). The study variants were classified as class 5 (pathogenic) o class 4 (likely pathogenic).

The FASTAQ datasets obtained from genetic sequencing were uploaded into the seven BI platforms for secondary and tertiary analysis of genomic data. Following an analysis of the tools currently available, the seven most popular AI-based tools in the sector were selected, namely: Emedgene (Illumina^®^, Inc, CA, USA.); eVai (enGenome, Pavia, Italy); Varsome Clinical (Saphetor SA, Lausanne, Switzerland); CentoCloud^®^ (Centogene GmbH, Rostock, Germany); and CLC genomics workbench for secondary analysis; and QIAGEN Clinical Insight (QCI) Interpret for tertiary analysis (Qiagen GmbH, Hilden, Germany); SeqOne (Montpellier, France) and Franklin (Genoox, Tel Aviv-Yafo, Israel). Apart from genomic data, patient phenotype was entered into each platform using HPO terms for further BI analysis (see Supplementary Material, Table 5).

First, the performance of each platform in prioritizing variants was evaluated. The position in which each program ranked the 24 causative variants was analyzed. When the variant ranked first, it was defined to be in the Top 1. When it ranked in the top five positions, it was defined as Top 5; as Top 10 if it was in the top 10 positions, and as Top 15 if it was ranked within the top 15 positions. When the variant was beyond position 16, it was defined as ‘not prioritized’ (NP). When the causal variant was not found in the total list of variants, it was defined as ‘not detected’ (ND). NP and ND variants were considered an ‘event’, which indicated a selection failure that may clearly prevent diagnosis. Secondly, the classification automatically assigned to the 24 variants by the platforms was compared. All platforms included in the study classify variants automatically according to the ACMG/AMP and ACMG/ClinGen guidelines [18], 19]. The level of agreement between the classification assigned by each platform and the classification of reference was assessed. In view that all variants were pathogenic and likely pathogenic, when a variant was classified as pathogenic instead of likely pathogenic and vice versa, it was identified as discordant without clinical implications. When a variant was classified as of uncertain significance and benign, it was identified as discordant with clinical implications.

## Results

### Evaluation of automated prioritization

**Figure 1:**
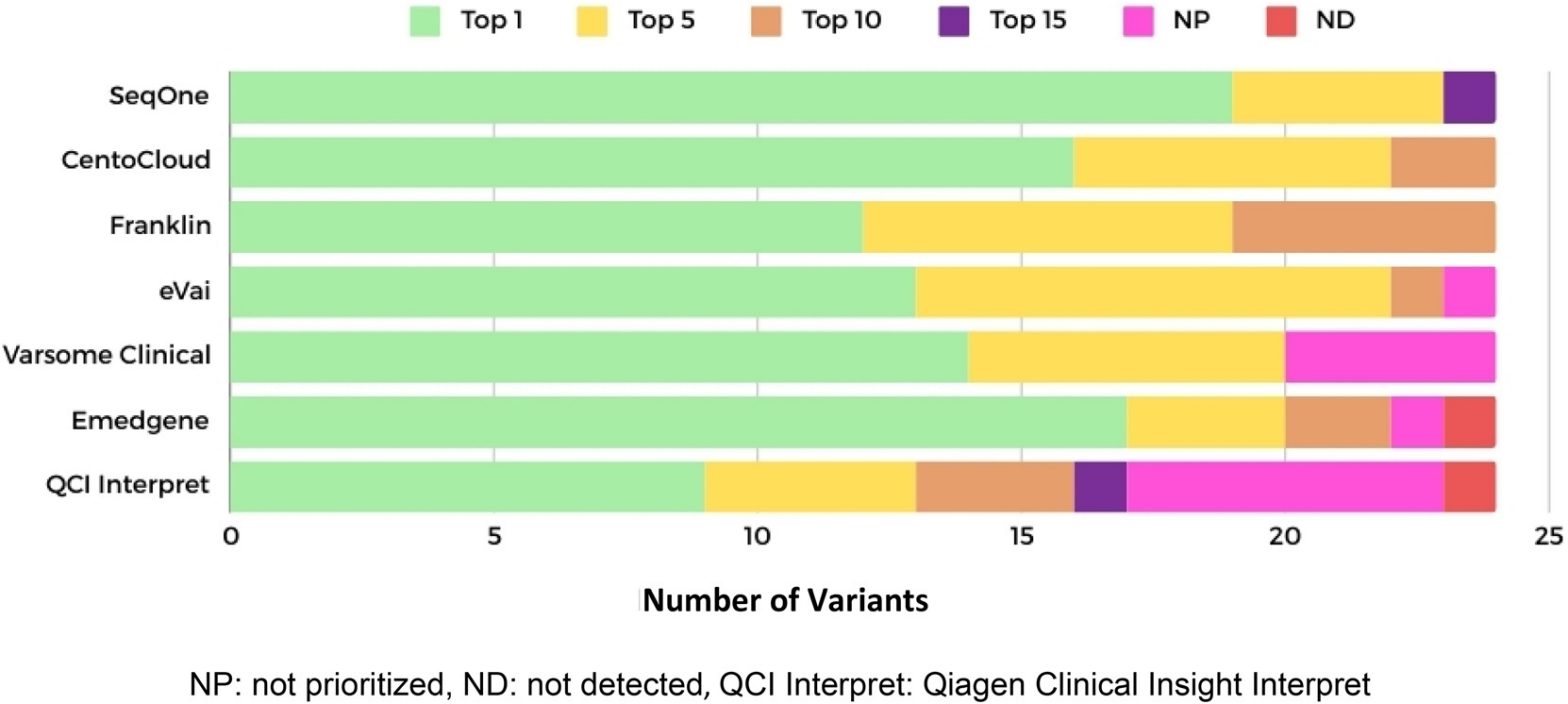
Ranking of platforms by in variant prioritization performance. NP: not prioritized, ND: not detected, QCI Interpret: Qiagen Clinical Insight Interpret.

The best platforms, ranking in the Top 1 and Top 5, were SeqOne, CentoCloud and eVai. Other platforms in the Top 10 were CentoCloud and Franklin (Figure 1).

**Figure 2:**
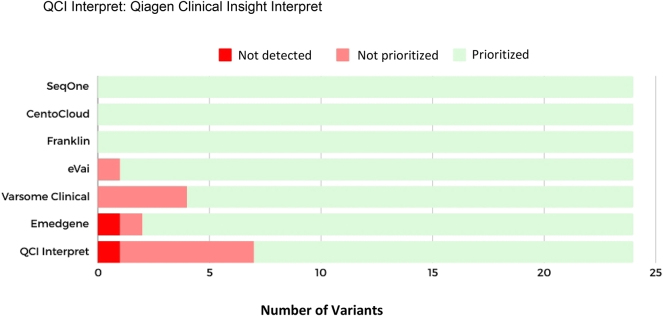
Concordances and discordances in automatic classification as compared against the classification of reference.

Emedgene ranked 22 variants (92 %) in the Top 10. However, this platform failed to prioritize a nonsense SNV in the *PAX9* gene of patient R14 and did not detect a CNV in patient R1 (see Supplementary Material, Table 6). Varsome Clinical failed to prioritize four variants (17 %), of which three were CNVs. The rest of variants, 14 (58 %), were ranked in the Top 1, and six (25 %) in the Top 5. The QCI Interpret yielded the poorest results, was the platform with the highest number of non-prioritized variants (six, 25 %) and failed to detect a SNV (4 %) in the *CDKL5* gene of patient R15 (see Supplementary Material, Table 7). By number of events, QCI Interpret was last in the ranking, with six non-prioritized variants and one not detected. Emedgene ranked sixth, as it failed to detect a variant and prioritize another. Varsome Clinical ranked fifth, as it failed to prioritize four variants. Finally, eVai ranked third, as it failed to prioritize a variant (Figure 2).

In total, 14 events were identified. Emedgene failed to detect CNV 2p16.3 (DEL). QCI Interpret did not detect the *CDKL5*:c.283-2A>G variant. Varsome Clinical had a poor performance in prioritizing CNVs, as it failed to prioritize three of the six CNVs evaluated. Five variants were not prioritized by QCI Interpret, of which two were missense, two were frameshift and one was a nonsense mutation (Table 1).

**Table 1:** Variants not detected and not prioritized.

Patient	Chromosomal position	Type of CNV	Event	Software
R1	2p16.3	Del	ND	Emedgene
			NP	VC, QCII
R4	15q11.2	Del	NP	VC
R9	Xp22.31	Del	NP	VC

**Patient**	**Gene**	**Variant**	**Event**	**Software**

R15	*CDKL5*	c.283-2A>G	ND	QCII
R1	*SPAST*	c.1617-2A>G	NP	VC
R8	*EXT1*	c.1037G>T	NP	QCII
R12	*CNOT1*	c.2071del	NP	QCII
R14	*PAX9*	c.554C>A	NP	QCII, Emedgene
R17	*BSND*	c.23G>A	NP	eVai, QCII
R19	*JAG1*	c.221_224del	NP	QCII

CNV, copy number variant; NP, not prioritized; ND, not detected; VC, Varsome Clinical; QCII, Qiagen Clinical Insight Interpret.

If the platforms that failed to prioritize or detect variants due to their clinical impact are excluded, SeqOne was the one with the highest performance. This platform ranked 19 variants in the Top 1, four in the Top 5 and one in the Top 15. The second with the best performance was CentoCloud, with 16 variants ranked in the Top 1, six in the Top 5, and two in the Top 10. Finally, Franklin ranked third, with 12 variants in the Top 1, seven in the Top 5 and five in the Top 10.

### Evaluation of automated prioritization

**Figure 3:**
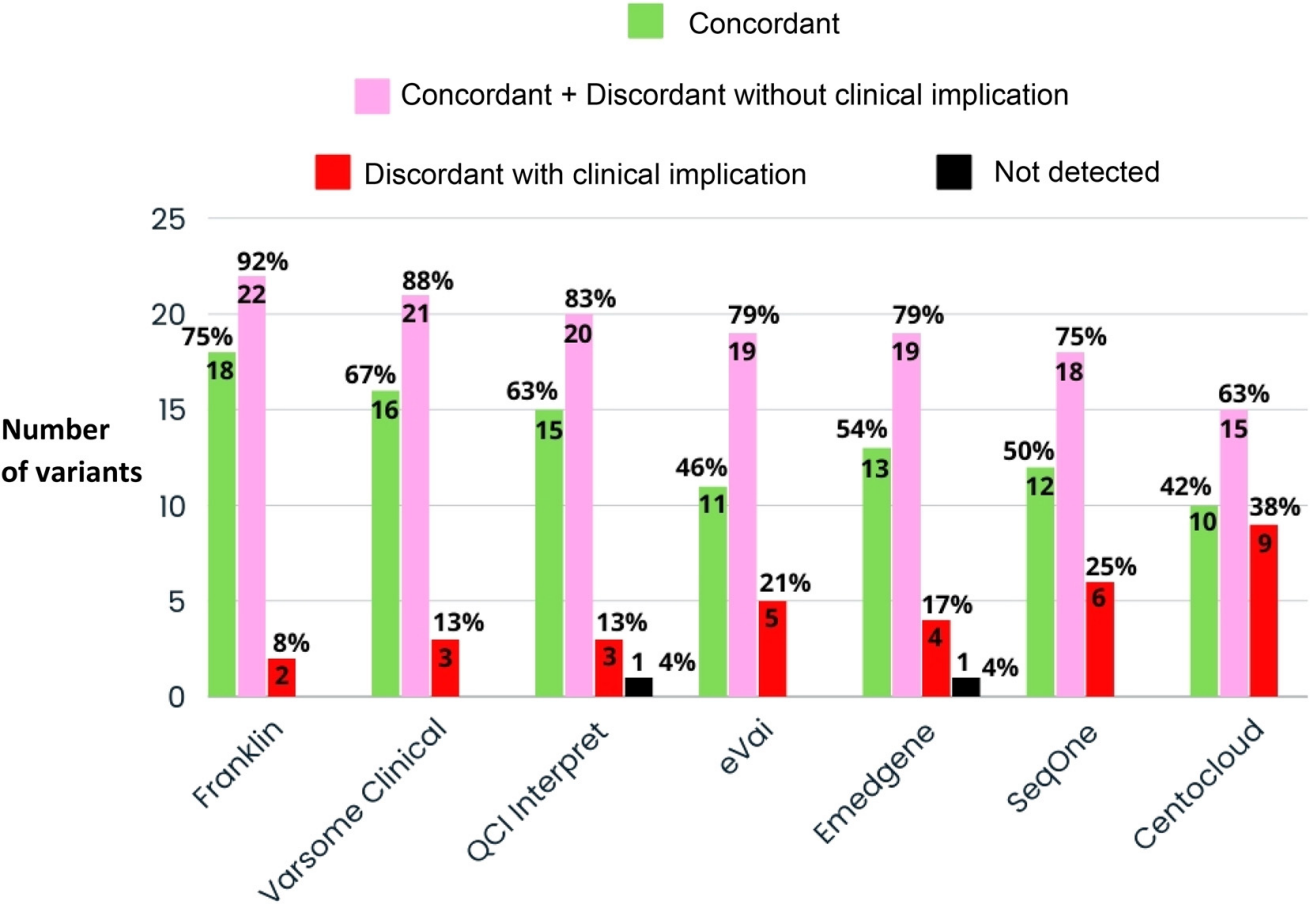
Comparative analysis of automatic classification vs. the reference classification. QCI Interpret: Qiagen Clinical Insight Interpret.

In general, all platforms performed a high-quality automated classification according to the guidelines available [18], 19]. The automated variant classification performed by Franklin was strictly concordant with the classification of reference in 18 of the 24 variants (75 %), followed by Varsome Clinical in 16 (67 %); QCI Interpret in 15 (63 %); Emedgene in 13 (54 %); SeqOne in 12 variants (50 %); eVai in 11 variants (46 %) and CentoCloud in 10 (42 %). Franklin performed an adequate classification (concordant with the reference classification and discordant without clinical implications) in 22 of the 24 variants (92 %), followed by Varsome Clinical in 21 variants (88 %); QCI Interpret in 20 variants (83 %); Emedgene and eVai in 19 variants (79 %); SeqOne in 18 variants (75 %); and CentoCloud in 15 variants (63 %). The platforms with the highest number of discordant classifications with clinical implications were CentoCloud, with nine of the 24 (38 %) variants with a discordant classification with clinical implications, eight of which were classified as variants of uncertain significance and one as benign. In contrast, Franklin yielded the lowest number of discordant classifications with clinical implications, only two of the 24 variants evaluated (8.3 %) (Figure 3).

**Figure 4:**
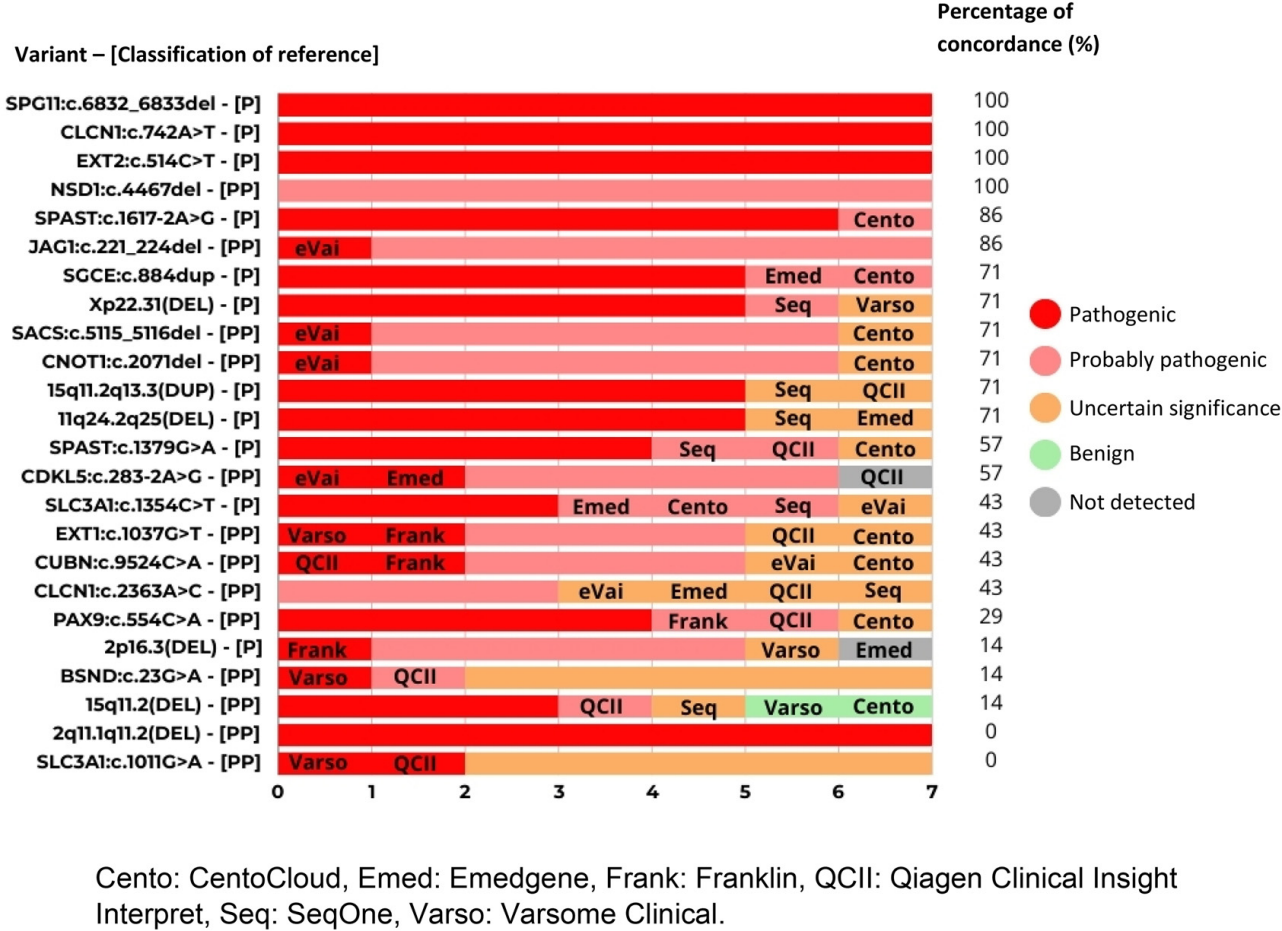
Comparative analysis of automatic classification by variant; concordance with the classification of reference; and discordant platforms. Cento: CentoCloud, Emed: Emedgene, Frank: Franklin, QCII: Qiagen Clinical Insight Interpret, Seq: SeqOne, Varso: Varsome Clinical.

Considering variants separately, all platforms agreed on the classification of only four variants (SPG*11*:c.6832_6833del, *CLCN1*:c.742A>T, *EXT2*:c.514C>T and *NSD1*:c.4467del) and were consistent with the classification of reference. In relation to the *SPAST*:c.1617-2A>G, *JAG1*:c.221_224del variants, there was a platform with a discordance without clinical implications. Concerning the *SGCE*:c.884dup and *CDKL5*:c.283-2A>G variants, there were two platforms with a discordance without clinical implications. Notably, all platforms were concordant and classified the 2q11.1q11.2 (DEL) variant as pathogenic, although it was classified as likely pathogenic in the classification of reference. In another 15 variants, a discrepancy with clinical implications was detected. Finally, whereas UCG-DUH and QCI Interpret classified the CNV 15q11.2 (DEL) as likely pathogenic, Emedgene, eVai and Franklin, classified it as pathogenic, SeqOne as a variant of uncertain significance, and CentoCloud and Varsome Clinical as a class 1 benign variant (Figure 4).

**Figure 5:**
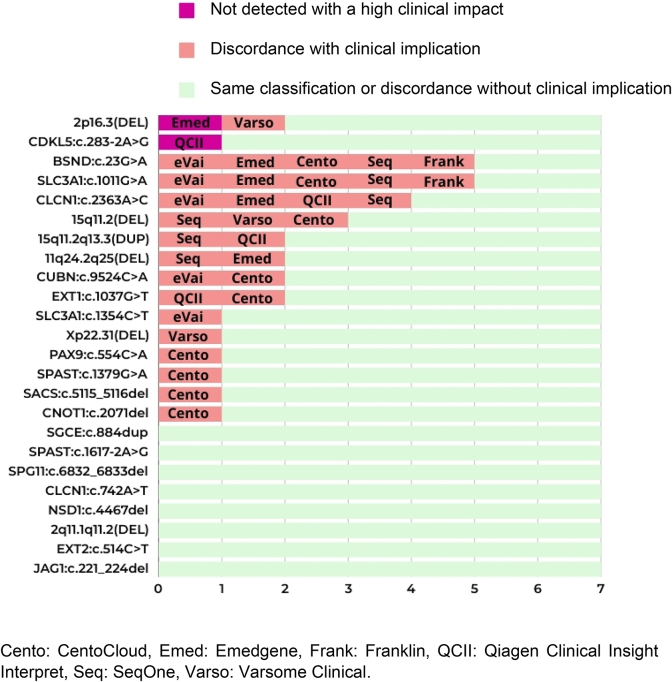
Concordances and discordances in automatic classification with respect to the classification of reference and the platforms analysed. Cento: CentoCloud, Emed: Emedgene, Frank: Franklin, QCII: Qiagen Clinical Insight Interpret, Seq: SeqOne, Varso: Varsome Clinical.

In total, there were 32 discrepancies with a potential clinical implication concerning correct diagnosis. Three or more platforms showed a discordance with clinical implications in relation to *BSND*:c.23G>A, *SLC3A1*:c.1011G>A, *CLCN1*:c.2363A>C and 15q11.2 (DEL) (Figure 5).

## Discussion

Technological advances in massive gene sequencing have contributed to the emergence of new applications to genetic diagnostics. These advances have increased diagnostic yield, reduced time to diagnosis and improved turnaround time. However, these new technologies have some drawbacks. High-throughput platforms generate large genomic datasets which interpretation is challenging. The processing of genomic data involves several BI processes. It is in the tertiary analysis after variant calling and annotation where the variant that may cause patient phenotype is identified. In the recent years, a variety of platforms have been developed to help professionals filter, prioritize, classify and interpret variants. Apart from containing automated classification modules based on guidelines, these platforms integrate AI-based algorithms for the automated prioritization of variants.

This is the first study to retrospectively evaluate a pilot group of patients to establish the criteria to validate the optimal tertiary analysis platform for daily laboratory practice.

Variant prioritization is essential in genetic diagnostics. Traditionally, this process was performed manually by using different filters to reduce the number of variants until the potential cause of the patient phenotype was identified. This manual approach was effective but time-consuming and complex, as it involved the analysis of large datasets of genomic data. Following filtering, variants were classified manually according to the criteria established in ACMG/AMP guidelines, among others.

In relation to process optimization, automated variant prioritization is crucial in improving the performance of genetic diagnostics. Automation significantly reduces turnaround time and enables the analysis of large genomic datasets, thereby leading to a more rapid and accurate identification of clinically relevant variants.

Automated classification is a step forward but stays in the background. Not all rules can be automated, since some require external information for them to be applied, such as the variant inheritance or familial disease segregation pattern. In the recent years, efforts have been made to standardize the application of rules. However, this step is still open to subjectivity. As a result, manual classification is not exempt from uncertainty. Some authors suggest the use of a quantitative approach to apply guidelines [32] i.e. specifications for the application of particular criteria [33] and specific gene guidelines such as the guidelines for the *APC*, *BRCA1* and *BRCA2* genes [34], 35].

Considering the results of our study for variant prioritization, SeqOne, CentoCloud and Franklin prioritized all variants, and eVai only failed to prioritize one of the 24 variants evaluated. Varsome Clinical demonstrated an acceptable prioritization capability, as it ranked 83 % of variants in the Top 5, but failed to prioritize four of the 24 variants, three of which were CNVs. Emedgene did not prioritize a *PAX9* variant, which is relevant, as it has deleterious effects. The nucleotide changes at position c.554, which transforms cytosine to adenine, generates a premature stop codon. This probably results in the absence or disruption of the protein. In addition, patient phenotype is very specific and is extensively documented for this gene. Emedgene also failed to detect a deletion in the *NRXN1* gene at chromosome 2p16.3. The deletion of two exons demonstrates the intrinsic limitation of the exome for detecting small CNVs, and the need for a CNV model for secondary analysis. QCI Interpret is fitted with a phenotype-driven ranking (PDR) that still needs further development. It is striking that a variant that ranks first and is classified as pathogeinc when PDR is applied disappears in the ocean of variants and is classified as a variant of uncertain significance. Apparently, the phenotype-driven ranking module of QCI Interpret is too strictly linked to the diseases described. This rigidity does not align adequately with patient phenotype. The evaluation of QCI Interpret for the 24 variants was carried out using the general list of variants. The PDR option was not used.

Franklin, QCI Interpret and Varsome Clinical demonstrated the best performance in automated variant classification. However, it is worth noting that the two latter failed to prioritize a significant number of variants. In contrast, albeit they perform a more conservative variant classification, CentoCloud, SeqOne and eVai prioritized variants by ranking them in the first positions but classified them as variants of uncertain significance, thereby requiring external clinical support.

In summary, a perfect platform is not currently available. An optimal platform must provide an automated variant prioritization without failing to detect any causal variant. Automated classification must also be accurate. The data obtained in this comparative study reveal the high performance of these platforms in variant prioritization resulting from the use of bioinformatics and AI-based algorithms.

These platforms are effective in general, with a notable performance in automated variant prioritization and classification. However, some differences highlight the relevance of appropriate prioritization for successful diagnosis. Although automated classification is a significant added value of these platforms, it can be modified and/or questioned by other critical information which the analysts and clinicians may have access to. These BI tools are constantly being improved, with enhanced capabilities to keep up to date with scientific advances. In general, the platforms evaluated do not only support but also optimize the tasks of clinical laboratory specialists and facilitate the implementation of WES in clinical practice.

A limitation of this study is that results are provided only for a small number of variants. For more consistent results, larger studies are needed that do not only include pathogenic and likely pathogenic variants, but also a selection of population variants. These studies would enable a more accurate evaluation of the automated genotyping and classification capabilities of each platform.

In conclusion, the Franklin platform showed a superior overall performance. With respect to automated prioritization, Franklin ranked the third, following SeqOne and CentoCloud. In terms of automated classification, Franklin showed the highest concordance with the classification of reference and has presented fewer discordances with clinical implications.

## Supplementary Material

Supplementary Material
