# Supplementary material for: Evaluating seven bioinformatics platforms for tertiary analysis of genomic data from whole exome sequencing in a pilot group of patients
Source: Adv Lab Med. 2025 Mar 10;6(1):28–36. doi: 10.1515/almed-2025-0031 (PMC11949535; doi:10.1515/almed-2025-0031)
Supplement: Supplementary file 1 — Supplementary Material [file j_almed-2025-0031_suppl_001.docx]

**Supplementary material Tables 1-7.**

**Abbreviatures:**

ACMG, American College of Medical Genetics and Genomics

AD, autosomal dominant

AMP, Association for Molecular Pathology

AR, autosomal recessive

B, benign

BP, break point

C, contiguous

Clas, classification

CNV, copy number variant

Del, deletion

Dup, duplication

F, female

Het, heterozygous

Hom, homozygous

HPO, Human Phenotype Ontology

Idic, isodicentric

Inv, inversion

kb, kilobase

LXD, X-linked dominant

m, moderate

M, male

NC, non-contiguous

ND, not detected

NP, not prioritized

OMIM, Online Mendelian Inherited in Man

P, pathogenic

PP, likely pathogenic

Pos, position

QCII, QiaGene Clinical Insight Interpret

Ref, reference

sp, supporting

st, strong

V, variant of uncertain significance

vst, very strong

**Table 1.** Reference CNV classification according to the criteria of clinical guidelines.

| Patient | Chromosomal position | GRCh38 Genomic Coordinates | Type of  CNV | Length kb | Clas  Ref | ACMG/ClinGene Criteria |
| --- | --- | --- | --- | --- | --- | --- |
| R1 | 2p16.3 | NC_000002.12:g.(51026361_51028284)del | Del | 1.9 | P | 2C(+0,9),4L(+0,15) |
| R4 | 2q11.1q11.2 | NC_000002.12:g.(95948520_96986324)del | Del | 1040 | PP | 2A(+0,9) |
|  | 15q11.2 | NC_000015.10:g.(22786647_23039554)del | Del | 253 | PP | 2A(+0,9) |
| R9 | Xp22.31 | NC_000023.11:g.(7049993_7350563)del | Del | 300 | P | 2A(+1) |
| R11 | 15q11.2q13.3 | NC_000015.10:g.(22786647_32158617)dup | Dup | 9370 | P | 2A(+1),3B(+0,45),4L(+0,15) |
| R20 | 11q24.2q25 | NC_000011.10:g.(127000445_134387705)del | Del | 7390 | P | 2A(+1),3B(+0,45),4L(+0,15) |

**Table2.** Reference SNV classification and small deletions and duplications according to the criteria of clinical guidelines.

| Patient | Gen | MANE Select Transcript | Variant | Protein Effect | Type of Variant | Zygosity | Clas  Ref | ACMG/AMP Criteria |
| --- | --- | --- | --- | --- | --- | --- | --- | --- |
| R1 | *SPAST* | NM_014946.4 | c.1617-2A>G | - | Splicing | Het | P | PVS1(vst);PM2(sp);PP5(sp) |
| R2 | *SPAST* | NM_014946.4 | c.1379G>A | p.Arg460His | Missense | Het | P | PM1(st);PM2(sp);PM5(m);PP3(sp);PP5(sp) |
| R3 | *SPG11* | NM_025137.4 | c.6832_6833del | p.Ser2278Leufs*61 | Frameshift | Hom | P | PVS1(vst);PM2(sp);PP1(sp);PP5(sp) |
| R5 | *CUBN* | NM_001081.4 | c.9524C>A | p.Ser3175* | Nonsense | Hom | PP | PVS1(vst);PM2(sp) |
| R6 | *CLCN1* | NM_000083.3 | c.742A>T | p.Lys248* | Nonsense | Het | P | PVS1(vst);PM2(sp);PM3(sp);PP5(sp) |
|  |  |  | c.2363A>C | p.Gln788Pro | Missense | Het | PP | PS3(st);PM2(sp);PM3(sp);PP5(sp) |
| R7 | *SLC3A1* | NM_000341.4 | c.1011G>A | p.Pro337= | Silent | Het | PP | PS3(st);PM2(sp);PM3(sp);PP5(sp) |
|  |  |  | c.1354C>T | p.Arg452Trp | Missense | Het | P | PM1(st);PM2(sp);PM3(sp);PM5(m);PP3(sp);PP5(sp) |
| R8 | *EXT1* | NM_000127.3 | c.1037G>T | p.Arg346Ile | Missense | Het | PP | PM2(sp);PM5(m);PP3(sp);PP5(m) |
| R10 | *SACS* | NM_014363.6 | c.5115_5116del | p.Ser1706Phefs*9 | Frameshift | Hom | PP | PVS1(vst);PM2(sp) |
| R12 | *CNOT1* | NM_016284.5 | c.2071del | p.Val691Leufs*13 | Frameshift | Het | PP | PVS1(vst);PM2(sp) |
| R13 | *NSD1* | NM_022455.5 | c.4467del | p.Asp1489Glufs*14 | Frameshift | Het | PP | PVS1(vst);PM2(sp) |
| R14 | *PAX9* | NM_001372076.1 | c.554C>A | p.Ser185* | Nonsense | Het | PP | PVS1(vst);PM2(sp) |
| R15 | *CDKL5* | NM_001323289.2 | c.283-2A>G | - | Splicing | Het | PP | PVS1(vst);PM2(sp) |
| R16 | *SGCE* | NM_003919.3 | c.884dup | p.Leu295Phefs*3 | Frameshift | Het | P | PVS1(vst);PM2(sp);PP5(sp) |
| R17 | *BSND* | NM_057176.3 | c.23G>A | p.Arg8Gln | Missense | Hom | PP | PM1(st);PM2(sp);PM5(m);PP3(sp);PP5(sp) |
| R18 | *EXT2* | NM_207122.2 | c.514C>T | p.Gln172* | Nonsense | Het | P | PVS1(vst);PM2(sp);PP5(sp) |
| R19 | *JAG1* | NM_000214.3 | c.221_224del | p.Tyr74Serfs*86 | Frameshift | Het | PP | PVS1(vst);PM2(sp) |

**Table 3 Patients with CNVs**

| Patient | Chromosomal position | GRCh38 Genomic Coordinates | Type of CNV | Number of copies | Length (kb) | Clas  Ref | ClinGen |
| --- | --- | --- | --- | --- | --- | --- | --- |
| R1 | 2p16.3 | NC_000002.12:g.(51026361_51028284)del | Deletion | 1 | 1,9 | P | Intragenic *NRXN1* deletion: complex neurodevelopmental disorder |
| R4 | 2q11.1q11.2 | NC_000002.12:g.(95948520_96986324)del | Deletion | 1 | 1040 | PP | Recurrent 2q11.2 deletion |
|  | 15q11.2 | NC_000015.10:g.(22786647_23039554)del | Deletion | 1 | 253 | PP | Recurrent 15q11.2 deletion (BP1-BP2) |
| R9 | Xp22.31 | NC_000023.11:g.(7049993_7350563)del | Deletion | 0 | 300 | P | Recurrent Xp22.31 deletion involving the *STS* gene associated with recessive X-linked ichthyosis |
| R11 | 15q11.2q13.3 | NC_000015.10:g.(22786647_32158617)dup | Duplication | 4 | 9370 | P | Tetrasomy 15q with high suspicion of marker chromosome inv dup (15) or idic(15) |
| R20 | 11q24.2q25 | NC_000011.10:g.(127000445_134387705)del | Deletion | 1 | 7390 | P | Terminal region 11q23q25 associated with Jacobsen Syndrome |

**Table 4.** Patients with SNVs and small deletions and duplications

| Patient | Gene | Transcript MANE Select | Genomic GRCh38 coordinate | Variant | Protein effect | Type of variant | Inheritance | Zygosity | Clas  Ref | OMIM | Condition |
| --- | --- | --- | --- | --- | --- | --- | --- | --- | --- | --- | --- |
| R1 | *SPAST* | NM_014946.4 | 2:32144935 | c.1617-2A>G | - | Splicing | AD | Het | P | #182601 | Autosomal dominant spastic paraplegia type 4 |
| R2 | *CongeniSPAST* | NM_014946.4 | 2:32136934 | c.1379G>A | p.Arg460His | Missense | AD | Het | P | #182601 | Autosomal dominant spastic paraplegia type 4 |
| R3 | *SPG11* | NM_025137.4 | 15:44566227 | c.6832_6833del | p.Ser2278Leufs*61 | Frameshift | AR | Hom | P | #604360 | Autosomal dominant spastic paraplegia type 11 |
| R5 | *CUBN* | NM_001081.4 | 10:16851374 | c.9524C>A | p.Ser3175* | Nonsense | AR | Hom | PP | #261100 | Imerslund-Grasbeck 1 syndrome |
| R6 | *CLCN1* | NM_000083.3 | 7:143323354 | c.742A>T | p.Lys248* | Nonsense | AR | Het | P | #255700 | Autosomal recessive myotonia |
|  |  |  | 7:143346657 | c.2363A>C | p.Gln788Pro | Missense |  | Het | PP |  |  |
| R7 | *SLC3A1* | NM_000341.4 | 2:44300090 | c.1011G>A | p.Pro337= | Silent | AR | Het | PP | #220100 | Cystinuria |
|  |  |  | 2:44312607 | c.1354C>T | p.Arg452Trp | Missense |  | Het | P |  |  |
| R8 | *EXT1* | NM_000127.3 | 8:117837127 | c.1037G>T | p.Arg346Ile | Missense | AD | Het | PP | #133700 | Hereditary multiple exostoses type 1 |
| R10 | *SACS* | NM_014363.6 | 13:23338760 | c.5115_5116del | p.Ser1706Phefs*9 | Frameshift | AR | Hom | PP | #270550 | Spastic ataxia of Charlevoix-Saguenay, |
| R12 | *CNOT1* | NM_016284.5 | 16:58560271 | c.2071del | p.Val691Leufs*13 | Frameshift | AD | Het | PP | **#**619033 | Vissers-Bodmer syndrome |
| R13 | *NSD1* | NM_022455.5 | 5:177246766 | c.4467del | p.Asp1489Glufs*14 | Frameshift | AD | Het | PP | #117550 | Sotos syndrome |
| R14 | *PAX9* | NM_001372076.1 | 14:36663446 | c.554C>A | p.Ser185* | Nonsense | AD | Het | PP | #604625 | Selective tooth agenesis-3 |
| R15 | *CDKL5* | NM_001323289.2 | X:18579846 | c.283-2A>G | - | Splicing | LXD | Het | PP | #300672 | Epileptic and developmental encephalopathy 2 |
| R16 | *SGCE* | NM_003919.3 | 7:94600798 | c.884dup | p.Leu295Phefs*3 | Frameshift | AD | Het | P | #159900 | Myoclonic dystonia 11 |
| R17 | *BSND* | NM_057176.3 | 1:54999209 | c.23G>A | p.Arg8Gln | Missense | AR | Hom | PP | #602522 | Sensorineural deafness with mild renal dysfunction |
| R18 | *EXT2* | NM_207122.2 | 11:44108226 | c.514C>T | p.Gln172* | Nonsense | AD | Het | P | #133701 | Hereditary multiple exostoses type 2 |
| R19 | *JAG1* | NM_000214.3 | 20:10672864 | c.221_224del | p.Tyr74Serfs*86 | Frameshift | AD | Het | PP | #118450 | Alagille 1 |

**Table 5.** Phenotype of each Patient in HPO terms.

| Patient | Sex | Términos HPO |
| --- | --- | --- |
| R1 | M | Global developmental delay HP:0001263; Delayed speech and language development HP:0000750; Drooling HP:0002307; Affected HP:0032320 |
| R2 | M | Gait disturbance HP:0001288; Spastic paraparetic gait HP:0031958; Intermittent painful muscle spasms HP:0011964; Affected HP:0032320 |
| R3 | F | Paraparesis HP:0002385; Cognitive impairment HP:0100543; Hypertelorism HP:0000316; Wide nasal bridge HP:0000431; Prominent nasal bridge HP:0000426; Specific learning disability HP:0001328; Affected HP:0032320 |
| R4 | M | Tall stature HP:0000098; Neurodevelopmental delay HP:0012758; Nevus of Ota HP:0009920; Unilateral cryptorchidism HP:0012741; Hypotonia HP:0001252; Autistic behavior HP:0000729; Affected HP:0032320 |
| R5 | M | Proteinuria HP:0000093; Abnormal urine protein level HP:0020129; Glomerular proteinuria HP:4000058; Affected HP:0032320 |
| R6 | M | Myotonia HP:0002486; Exercise-induced muscle stiffness HP:0008967; Myotonia of the upper limb HP:0012903; Myotonia of the lower limb HP:0012902; Affected HP:0032320 |
| R7 | F | Cystinuria HP:0003131; Increased sulfur amino acid level in urine HP:0033095; Aminoaciduria HP:0003355; Affected HP:0032320 |
| R8 | M | Short stature HP:0004322; Genu valgum HP:0002857; Osteochondrosis HP:0040188; Chronic kidney disease HP:0012622; Affected HP:0032320 |
| R9 | M | Ichthyosis HP:0008064; Dry skin HP:0000958; Scaling skin HP:0040189; Affected HP:0032320 |
| R10 | F | Dystonia HP:0001332; Chorea HP:0002072; Spasticity HP:0001257; Tip-toe gait HP:0030051; Clumsiness HP:0002312; Dysmetria HP:0001310; Babinski sign HP:0003487; Hyperreflexia HP:0001347; Clonus HP:0002169; Unsteady gait HP:0002317 Affected HP:0032320 |
| R11 | M | Infantile axial hypotonia HP:0009062; Limb hypertonia HP:0002509; Neurodevelopmental delay HP:0012758; Affected HP:0032320 |
| R12 | F | Intellectual disability HP:0001249; Coloboma HP:0000589; Esotropia HP:0000565; Specific learning disability HP:0001328; Abnormal social behavior HP:0012433; Language impairment HP:0002463; Affected HP:0032320 |
| R13 | F | Retrognathia HP:0000278; 2-3 finger syndactyly HP:0001233; Short neck HP:0000470; Microphthalmia HP:0000568; Narrow mouth HP:0000160; Global developmental delay HP:0001263; Atypical behavior HP:0000708; Micrognathia HP:0000347 Wide nasal bridge HP:0000431; Epicanthus HP:0000286; Long philtrum HP:0000343; Aggressive behavior HP:0000718; Tall stature HP:0000098; Recurrent respiratory infections HP:0002205; Affected HP:0032320 |
| R14 | M | Low-set ears HP:0000369; Agenesis of incisor HP:0006485; Protruding ear HP:0000411; Affected HP:0032320 |
| R15 | F | Neurodevelopmental delay HP:0012758; Hypotonia HP:0001252; Seizure HP:0001250; Affected HP:0032320 |
| R16 | F | Gait disturbance HP:0001288; Myoclonus HP:0001336; Delayed speech and language development HP:0000750; Language impairment HP:0002463; Dystonia HP:0001332; Hypertonia HP:0001276; Affected HP:0032320 |
| R17 | F | Retrognathia HP:0000278; Impulsivity HP:0100710; Microphthalmia HP:0000568; Astigmatism HP:0000483; Atypical behavior HP:0000708; Hypermetropia HP:0000540; Sensorineural hearing impairment HP:0000407; Microcephaly HP:0000252 Intellectual disability HP:0001249; Affected HP:0032320 |
| R18 | F | Multiple exostoses HP:0002762; Multiple enchondromatosis HP:0005701; Osteochondroma HP:0030431; Affected HP:0032320 |
| R19 | M | Mandibular prognathia HP:0000303; Partial anomalous pulmonary venous return HP:0010773; Butterfly vertebrae HP:0003316; Prominent forehead HP:0011220; Triangular face HP:0000325; Cholestasis HP:0001396; Small for gestational age HP:0001518; Intrauterine growth retardation HP:0001511; Patent foramen ovale HP:0001655; Abnormal calvaria morphology HP:0002683; Jaundice HP:0000952; Affected HP:0032320 |
| R20 | F | Intellectual disability HP:0001249; Gait disturbance HP:0001288; Broad-based gait HP:0002136; Frequent falls HP:0002359; Upper motor neuron dysfunction HP:0002493; Seizure HP:0001250; Hypomimic face HP:0000338; Camptocormia HP:0100595; Parkinsonism HP:0001300; Rigidity HP:0002063; Asterixis HP:0012164; Ataxia HP:0001251; Brisk reflexes HP:0001348; Impaired vibratory sensation HP:0002495; Bradykinesia HP:0002067; Lower limb muscle weakness HP:0007340; Dysmetria HP:0001310; Hypointensity of cerebral white matter on MRI HP:0007103; Abnormal cerebral vascular morphology HP:0100659; Leukoencephalopathy HP:0002352; Cranial hyperostosis HP:0004437; Affected HP:0032320 |

| Patient | Chromosomal position | Type of CNV | Length kb | Clas  Ref | Emedgene | | eVai | | Varsome Clinical | | Centocloud | | QCII | | SeqOne | | Franklin | |
| --- | --- | --- | --- | --- | --- | --- | --- | --- | --- | --- | --- | --- | --- | --- | --- | --- | --- | --- |
|  |  |  |  |  | Clas | Pos | Clas | Pos | Clas | Pos | Clas | Pos | Clas | Pos | Clas | Pos | Clas | Pos |
| R1 | 2p16.3 | Del | 1,9 | P | - | ND | PP | 5 | V | NP | PP | 1 | PP | NP | PP | 1 | P | 10 |
| R4 | 2q11.1q11.2 | Del | 1040 | PP | P | 10 | P | 5 | P | 1 | P | 1 | P | 1 | P | 1 | P | 10 |
|  | 15q11.2 | Del | 253 | PP | P | 1 | P | 5 | B | NP | B | 10 | PP | 5 | V | 5 | P | 10 |
| R9 | Xp22.31 | Del | 300 | P | P | 1 | P | 10 | V | NP | P | 1 | P | 1 | PP | 1 | P | 5 |
| R11 | 15q11.2q13.3 | Dup | 9370 | P | P | 1 | P | 1 | P | 1 | P | 1 | V | 1 | V | 1 | P | 5 |
| R20 | 11q24.2q25 | Del | 7390 | P | V | 1 | P | 5 | P | 1 | P | 1 | P | 1 | V | 1 | P | 10 |

**Table 6.** Comparison of the classification and positioning in the prioritization ranking of patients with CNVs across platforms.

| Patient | Gen | Variant | Protein effect | Clas Ref | Emedgene | | eVai | | Varsome Clinical | | Centocloud | | QCII | | SeqOne | | Franklin | | |
| --- | --- | --- | --- | --- | --- | --- | --- | --- | --- | --- | --- | --- | --- | --- | --- | --- | --- | --- | --- |
|  |  |  |  |  | Clas | Pos | Clas | Pos | Clas | Pos | Clas | Pos | Clas | Pos | Clas | Pos | Clas | Pos |  |
| R1 | *SPAST* | c.1617-2A>G | - | P | P | 1 | P | 1 | P | NP | PP | 5 | P | 1 | P | 1 | P | 10 |  |
| R2 | *SPAST* | c.1379G>A | p.Arg460His | P | P | 5 | P | 1 | P | 1 | V | 1 | PP | 1 | PP | 1 | P | 1 |  |
| R3 | *SPG11* | c.6832_6833del | p.Ser2278Leufs*61 | P | P | 1 | P | 1 | P | 1 | P | 1 | P | 1 | P | 1 | P | 1 |  |
| R5 | *CUBN* | c.9524C>A | p.Ser3175* | PP | PP | 1 | V | 1 | PP | 1 | V | 1 | P | 10 | PP | 1 | P | 1 |  |
| R6 | *CLCN1* | c.742A>T | p.Lys248* | P | P | 5 | P | 5 | P | 1 | P | 5 | P | 5 | P | 1 | P | 5 |  |
|  |  | c.2363A>C | p.Gln788Pro | PP | V | 1 (C) | V | 5 (NC) | PP | 5 (C) | PP | 5 (C) | V | 10 (C) | V | 5 (C) | PP | 1 (C) |  |
| R7 | *SLC3A1* | c.1011G>A | p.Pro337= | PP | V | 1 | V | 5 | P | 5 | V | 5 | P | 5 | V | 5 | V | 5 |  |
|  |  | c.1354C>T | p.Arg452Trp | P | PP | 5 (C) | V | 5 (C) | P | 1 (C) | PP | 5 (C) | P | 1 (C) | PP | 1 (NC) | P | 1 (C) |  |
| R8 | *EXT1* | c.1037G>T | p.Arg346Ile | PP | PP | 1 | PP | 1 | P | 1 | V | 1 | V | NP | PP | 1 | P | 1 |  |
| R10 | *SACS* | c.5115_5116del | p.Ser1706Phefs*9 | PP | PP | 1 | P | 5 | PP | 1 | V | 1 | PP | 15 | PP | 1 | PP | 1 |  |
| R12 | *CNOT1* | c.2071del | p.Val691Leufs*13 | PP | PP | 1 | P | 1 | PP | 5 | V | 1 | PP | NP | PP | 5 | PP | 1 |  |
| R13 | *NSD1* | c.4467del | p.Asp1489Glufs*14 | PP | PP | 1 | PP | 1 | PP | 1 | PP | 1 | PP | 10 | PP | 1 | PP | 1 |  |
| R14 | *PAX9* | c.554C>A | p.Ser185* | PP | P | NP | P | 1 | P | 1 | V | 5 | PP | NP | P | 1 | PP | 5 |  |
| R15 | *CDKL5* | c.283-2A>G | - | PP | P | 1 | P | 1 | PP | 5 | PP | 1 | - | ND | PP | 1 | PP | 5 |  |
| R16 | *SGCE* | c.884dup | p.Leu295Phefs*3 | P | PP | 1 | P | 1 | P | 5 | PP | 1 | P | 1 | P | 1 | P | 1 |  |
| R17 | *BSND* | c.23G>A | p.Arg8Gln | PP | V | 10 | V | NP | P | 1 | V | 10 | PP | NP | V | 15 | V | 5 |  |
| R18 | *EXT2* | c.514C>T | p.Gln172* | P | P | 1 | P | 1 | P | 1 | P | 1 | P | 5 | P | 1 | P | 1 |  |
| R19 | *JAG1* | c.221_224del | p.Tyr74Serfs*86 | PP | PP | 1 | P | 1 | PP | 5 | PP | 1 | PP | NP | PP | 1 | PP | 1 |  |

**Table7.** Comparison across platforms in the classification and positioning in the prioritization ranking of patients with SNVs and small deletions and duplications.
